# Incidence and Treatments of Bovine Mastitis and Other Diseases on 37 Dairy Farms in Wisconsin

**DOI:** 10.3390/pathogens11111282

**Published:** 2022-11-01

**Authors:** Juliano L. Gonçalves, Juliana L. de Campos, Andrew J. Steinberger, Nasia Safdar, Ashley Kates, Ajay Sethi, John Shutske, Garret Suen, Tony Goldberg, Roger I. Cue, Pamela L. Ruegg

**Affiliations:** 1 Department of Large Animal Clinical Sciences, College of Veterinary Medicine, Michigan State University, East Lansing, MI 48864, USA; 2 Department of Bacteriology, University of Wisconsin, Madison, WI 53706, USA; 3 Department of Animal Science, Macdonald Campus, McGill University, Montreal, QC H9X 3V9, Canada

**Keywords:** antibiotic, antimicrobial, dairy, disease, epidemiology

## Abstract

The aim of this research was to describe the incidence and treatments of mastitis and other common bovine diseases using one year of retrospective observational data (n = 50,329 cow-lactations) obtained from herd management software of 37 large dairy farms in Wisconsin. Incidence rate (IR) was defined as the number of first cases of each disease divided by the number of lactations per farm. Clinical mastitis (CM) remains the most diagnosed disease of dairy cows. Across all herds, the mean IR (cases per 100 cow-lactations) was 24.4 for clinical mastitis, 14.5 for foot disorders (FD), 11.2 for metritis (ME), 8.6 for ketosis (KE), 7.4 for retained fetal membranes (RFM), 4.5 for diarrhea (DI), 3.1 for displaced abomasum (DA), 2.9 for pneumonia (PN) and 1.9 for milk fever (MF). More than 30% of cows that had first cases of CM, DA, RFM, DI, and FD did not receive antibiotics. Of those treated, more than 50% of cows diagnosed with PN, ME and CM received ceftiofur as a treatment. The IR of mastitis and most other diseases was greater in older cows (parity ≥ 3) during the first 100 days of lactation and these cows were more likely to receive antibiotic treatments (as compared to younger cows diagnosed in later lactation). Cows of first and second parities in early lactation were more likely to remain in the herd after diagnosis of disease, as compared to older cows and cows in later stages of lactation. Most older cows diagnosed with CM in later lactation were culled before completion of the lactation. These results provide baseline data for disease incidence in dairy cows on modern U.S. dairy farms and reinforce the role of mastitis as an important cause of dairy cow morbidity.

## 1. Introduction

In the United States, milk production has continually increased as farms have intensified dairy management systems [1]. As farms have grown, the economic and animal welfare significance of important bovine diseases has been magnified [2]. Most dairy farms that milk more than 200 dairy cows utilize computer-based records, which can be used to perform epidemiologic evaluations of bovine diseases [3]. However, in contrast to standardized sources of data, definitions, detection systems and recording of health events are user-defined and can vary among farms [3]. Validation of animal health data is essential to avoid detection and misclassification biases that complicate comparisons among farms.

Incidence rates (IR) evaluate the first case of disease and are the best measure of disease risks and of the effectiveness of management practices focused on prevention. The IR of common diseases of dairy cows have varied among studies depending on year, data source and herd structure [1,2,3]. A nationally representative survey of disease occurrence on US dairy farms [1] reported that clinical mastitis (CM) was the most common disease and affected about 25% of cows per herd per year. Lameness (LA), metritis (ME), retained placenta (retained fetal membranes; RFM), and ketosis affected 17%, 7%, 5% and 4% of lactating cows per herd per year, respectively. Fewer animals (2–3%) experienced diarrhea (DI), pneumonia (PN), milk fever (MF) and displaced abomasum (DA). Occurrence of diseases results in reduced quality and production of milk, reduced fertility, and increased treatment costs [4,5,6,7]. Diseases also increase risks of death and culling thus reducing productive life [8,9].

Antimicrobials are often used for treatment of bacterial diseases, even when not all cases will benefit [10,11,12]. Mastitis is the most common bacterial disease of mature cows and is the most common reason that antibiotics are given. At least half of cases of non-severe clinical mastitis do not benefit from antimicrobial treatment and selective use of antimicrobials based on identification of etiological agents as well as cow-level factors should be recommended when possible for these cases [13,14].

Benchmarking diseases of dairy cows is useful as management practices and farm structures are modified and risk factors for disease evolve. Some risk factors such as parity (i.e., older cows) and the post parturient period are well known to increase risk of some diseases while season, farm size and level of milk production have known associations with specific diseases [15]. In recent years, U.S. farm management systems have changed dramatically with much larger farms now producing most of the milk and the incidence of common dairy diseases on large U.S. farms has not been reported for this group of herds. The objective of this observational study was to describe incidence and treatments of mastitis and other common bovine diseases on 37 large dairy farms in Wisconsin, USA.

## 2. Materials and Methods

### 2.1. Farm Eligibility, and Data Collection

Information about enrollment, eligibility and selection of dairy farms has been previously described [11]. In brief, recruitment letters were sent to all Wisconsin dairy farms, that contained ≥ 250 lactating dairy cows. Herds were eligible if they indicated that they recorded at least 90% of animal health events in their dairy record system and follow-up phone calls were used to confirm that health animal events were recorded. As part of the original study, farms were visited by researchers to review animal health records, validate disease definitions and recording criteria and collect additional information from farm workers who were responsible for animal care. Electronic record backup files were sent before our farm visits and researchers reviewed and printed all potential animal health events which were then discussed during the farm visit (which included a 2 h interview for which each farmer received $100 honorarium in recognition of the value of their time). During the interview researchers asked standardized questions about how each disease was defined, detected and recorded. Of farms (n = 40) included in the original study, data from 37 herds that recorded animal health events in a common dairy management software program (Dairy Comp305, Valley Agricultural Software, Tulare, CA, USA) were used in this study. Computerized health records and demographic data were retrieved for all lactations that commenced between August 2016 and August 2017 and supplemented, with additional information collected by researchers during a farm visit [11]. Datafiles included one year of retrospective observational data containing 50,329 cow-lactations.

### 2.2. Disease Definitions and Metrics

Cases were identified, treated, and recorded according to farm-specific criteria and represent farmer identified and recorded cases which reflect the perceptions and disease detection ability on well-managed commercial farms. The dairy records system is very flexible and farmers are allowed to define their own codes for entries. Prior to the farm visits, electronic record backup were obtained from the farm, and researchers printed all codes that were associated with animal health. During the farm visit, farm workers responsible for animal care were interviewed to ensure that disease codes were properly interpreted. When codes used to record events varied among farms, they were combined as appropriate. For example, user-defined events (UDE) were coded as CM when ‘MAST’, ‘MASTNT’, ‘MASTSYS’, ‘MASTTRT’, ‘MAST_LG’ and ‘MAST_MD’ events were recorded in the herd management software. Similarly, foot disorders (FD) included events coded as: ‘LAME’, ‘WRAP’, ‘HOOFROT’ and ‘FOOTROT’; metritis included events coded as: ‘MET’, ‘METR’, ‘METRIT’ and ‘UTERUS’; retained fetal membranes (retained placenta) included events coded as: ‘RP’ and ‘RETAINP’; ketosis included events coded as: ‘KET’, ‘KETOSIS’, ‘KET_LG’, ‘KET_MED’ and ‘KET_SML’; diarrhea included events coded as: ‘DIARRH’, ‘DIARHEA’, ‘DIARRHE’ and ‘DIAR’; pneumonia included events coded as: ‘PNEU’ and ‘PNEUM’; milk fever included events coded as: ‘MF’, ‘MF1’, ‘MF2’, ‘MFEVER’, ‘MILKFEV’, ‘MILKFVR’, ‘MLKFEVR’ and ‘MLKFVR’; and displaced abomasum included events coded as: ‘DA’, ‘RDA’ and ‘LDA.’ All farms recorded abortions as ‘ABORT’, death as ‘DIED’, calving as ‘FRESH’, pregnant as ‘PREG’ and ‘SOLD’ for culled.

Retrospective analysis of the incidence rate was defined as the number of first cases of each disease divided by the number of lactations (×100) [2]. Each cow was used in the numerator only once per disease per lactation. All lactations completed or terminated (drying off, death, or culling) within 522 days after initiation were used in the analysis. This limitation was the 99th percentile for lactation length and was used to exclude abnormally long lactations. The denominator used to calculate the IR of metritis included only non-pregnant milking cows within 14 days of calving.

### 2.3. Diseases Events and Treatment Remarks

Using the command ‘EVENTS\2SI ID DIM LACT FOR LACT>0’ in the Dairy Comp305 program [3], a total of 724,946 events were retrieved. From these, 19 events were excluded due to days in milk < 0. Events were further limited to those occurring between 0 and 522 days in milk (DIM) which excluded an additional 10,493 events. Selected non-health related events (such as pen moves) were also excluded (n = 244,488). The remaining events (n = 469,946) included all UDE related to disease (CM, ME, RFM, KE, DA, MF, LA, PN and DI; n = 45,163), and additional non-disease demographic events including dates of calving, culling, dry-off or death (n = 424,783).

A subset of data containing 36,374 UDE was created by including only first cases of each disease for each cow. All health records (including data from non-treated events) were reviewed to evaluate disease definitions. The final dataset contained 35,729 UDE, truncated to the 99th percentile of DIM at occurrence of each disease to exclude spurious events (e.g., a case of ketosis from a cow recorded as being 511 DIM). Based on physiological expectations, we defined cases to limit the maximum days in milk for occurrence of ME (16 d), RFM (10 d), KE (60 d) and MF (11 d) [2]. Treatment data was obtained as described in a previous study [11].

### 2.4. Statistical Procedures

The UDE was the unit of analysis. Descriptive statistics were used to verify data accuracy, detect missing data, and observe frequency distributions. All statistical analyses were performed using SAS version 9.4 (SAS Institute, Cary, NC, USA) and statistical significance was defined as *p* ≤ 0.05. Descriptive statistics were used to characterize farms and summarize IR of diseases and treatments. Separate analyses were performed using PROC GLIMMIX binomial or binary regression for the following dependent variables: (i) IR of each disease (binomial; number of first cases/number of lactations), (ii) use of antimicrobial treatments for each disease (binary; 0 = no treatment, and 1 = antimicrobial treatment), and (iii) proportion of cows remaining in the herd after occurrence of a disease (binary; 0 = culled or died, and 1 = cow remained in herd). The form of the generalized linear mixed model was:

Y_ijkmpq_ = µ + Bulk tank SCC (BTSCC)_i_ + Rolling herd average milk (RHA)_j_ + herd_ijk_ + parity_m_ + stage_p_ + season_q_ + *e*_ijkmpq_

For each disease (CM, ME, RFM, KE, FD, DA, PN, MF, DI), Y was either (i) binary response of having disease (Yes, No), (ii) received antimicrobial (Yes, No), or (iii) cows retained in herd after a disease (Yes, No). Dependent variables were binomial responses, analyzed using SAS PROC GLIMMIX. The independent variables were fixed effects of parity group (m = 1, 2, ≥3), stage of lactation at time of diagnosis (early ≥ 100 DIM, mid = >100 to ≤200 DIM, late ≥ 200 DIM), season of disease occurrence (summer was June to August; fall was September to November; winter was December to February; spring was March to May), and herd-level variable: BTSCC (<150,000, ≥150,000 cells/mL), RHA for milk (≤13,200, >13,200 kg per cow per year). All models included the random effect of herd nested within BTSCC and RHA. Backward selection was used to select variables that remained in the final models. Parity group and stage of lactation were forced in all models. The best models were selected, based on convergence and model fit (−2 log-likelihood and generalized chi square/df). Estimated regression coefficients of the models were exponentiated and interpreted as odds ratios.

## 3. Results

### 3.1. Farm Characteristics

Characteristics of the 40 farms enrolled in the original study have been previously described [11]. For herds (n = 37) used in this analysis, RHA was 13,377 (±164.4) kg of milk per cow per year and ranged from 10,829 to 15,059 kg (Supplementary Table S1). Farms contained 50,329 cow-lactations and the distribution of cows by parity group was: 37.8% first parity, 29.8% second parity and 32.4% of three or more parities. Average BTSCC was 142,600 (±7226 cells per mL) and ranged from 77,000 to 320,000 cells/mL.

Of 35,729 UDE, CM occurred most frequently (13,997 UDE occurring on 37 farms), followed by: LA (5176 UDE from 30 farms), ME (4948 UDE from 33 farms), RFM (3538 UDE from 35 farms), KE (3090 UDE from 30 farms), DA (1648 UDE from 37 farms), PN (1277 UDE from 35 farms), DI (1126 UDE from 17 farms) and MF (929 UDE from 32 farms). Among all UDE, 17,872 were not treated with antimicrobials while 17,857 had records of antimicrobial treatment. Among cows that exited the herd the greatest proportion were culled (31.9%; mean = 433 cow per farm, ranging from 17.2% to 44.1%) as compared to cows that died or were euthanized (4.9%; mean = 67 cow per farm, ranging from 0% to 21.1%).

### 3.2. Incidence Rate of Disease

**Figure 1:**
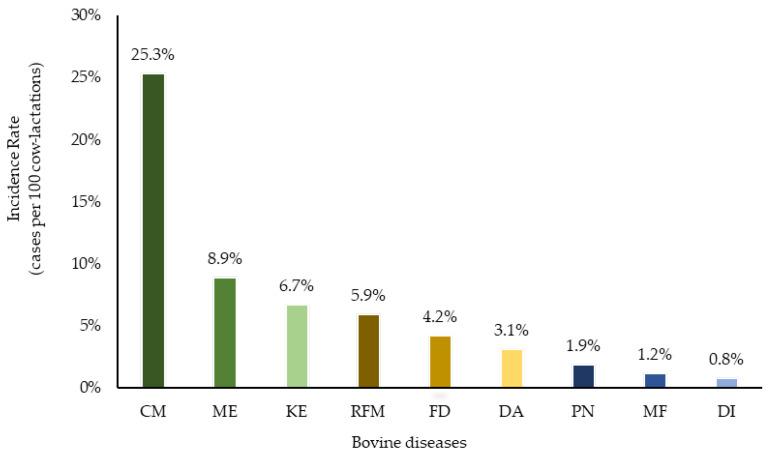
The median incidence rate (cases per 100 cow-lactations) of bovine diseases from 37 WI dairy herds in 2017. Incidence rate was defined as the number of first cases of each disease divided by the number of lactations per farm. CM = clinical mastitis; ME = metritis; KE = ketosis; RFM = retained fetal membranes; FD = foot disorders; DA = displaced abomasum; PN = pneumonia; MF = milk fever and DI = diarrhea.

Clinical mastitis was recorded on all 37 farms and the mean IR of CM was 24.4 (median = 25.3) cases per 100 cow-lactations, ranging from 1.7 (5th percentile) to 46.8 (95th percentile) (Supplementary Table S1 and Figure 1). Among farms, 81% recorded at least one case of FD while the mean IR was 14.5 (median = 4.2) cases per 100 cow-lactations, ranging from 0.1 to 57.7 (Supplementary Table S1). All farms reported uterine disorders (ME, RFM or uterine prolapse), while 89.2% recorded at least one ME event and 94.6% recorded RFM. The IR of ME was 11.2 (median = 8.9) cases per 100 cow-lactations ranging from 0.8 to 29.5. The mean IR of RFM was 7.4 (median = 5.9) cases per 100 cow-lactations, ranging from 0.8 to 15.8. The mean IR of KE was 8.6 (median = 6.7) cases per 100 cow-lactations, ranging from 0.2 to 31.5 and this disease was observed in 30 out of 37 farms (81.1%). Four other diseases (DI, DA, PN and MF) occurred less frequently (<5 cases per 100 cow-lactations). The mean IR were 4.5 (median of 0.8), 3.1 (median of 3.1), 2.9 (median of 1.9) and 1.9 (median of 1.2) for DI, DA, PN and MF, respectively.

### 3.3. Disease Specific Antibiotic Treatments

Most antimicrobials used for treatment of adult cows were formulations of ceftiofur (Table 1). For UDE that received antimicrobials, systemic treatments with formulations of ceftiofur were used to treat more than 65% of cases of PN and ME, while most CM were treated using intramammary (IMM) ceftiofur and a small proportion received systemic treatments. Ampicillin was the second most frequently used antimicrobial and was given to most cases of DA and less frequently for other diseases. Other antimicrobial classes included tetracyclines, sulfonamides, macrolides, lincosamides, amphenicol and aminoglycosides. About one-third of CM cases did not receive antibiotics. For the remaining diseases, no antimicrobial therapy was observed for 93.3% of FD cases, 79.0% of DI, 52.6% of RFP, 30.3% of DA, 12.5% of ME and 9.8% of PN cases. As expected, no antimicrobials were used to treat KE or MF.

### 3.4. Association of Incidence Rate of Disease with Parity and Stage of Lactation

The IR of CM, FD, RFM, KE, and MF was greater for cows in parity ≥3 as compared to cows in second or first parities (Table 2; *p* < 0.001). Second lactation cows had similar IR of PN and DA to older cows, and both had greater IR than first-parity cows. Metritis was the only disease that occurred more frequently in first parity cows followed by older cows and second parity. There was no association of parity with DI (*p* = 0.179).

The IR of CM, DA, DI and PN were greater in early lactation, but did not differ between mid and late lactation (Table 2). Foot disorders occurred more frequently in late lactation (2.7 cases per 100-cow-years) in comparison to similar IR for cows in early and mid-lactation (1.8 cases per 100-cow-years). Stage of lactation was not accounted for in the models for ME, RFM, MF and KE as case definitions limited DIM for these events. Farm size, BTSCC, season and RHA were not associated with IR of any diseases (*p* > 0.433).

**Figure 2:**
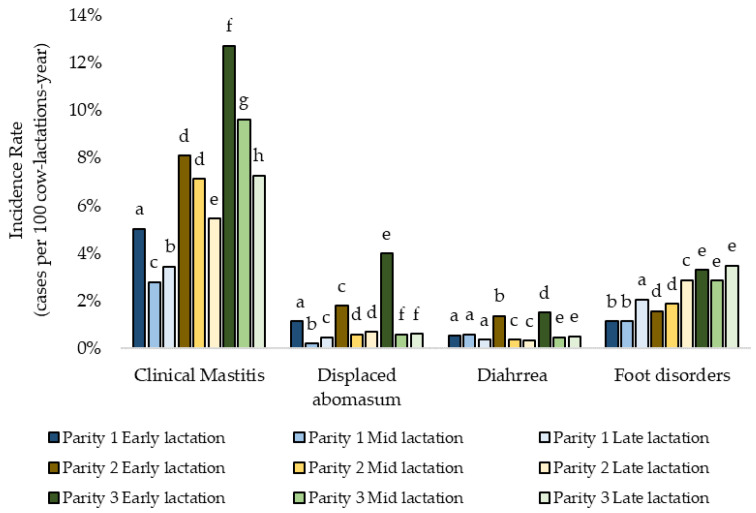
Incidence rate of diseases (clinical mastitis, displaced abomasum, diarrhea and foot disorders) with interaction of parity and stage of lactation for 37 WI dairy herds in 2017. Different superscripts letters (e.g., a–h) indicate comparisons among stages of lactation within parity and disease were significant (*p* < 0.004).

For four diseases (CM, FD, DA, and DI) an interaction of parity and stage of lactation was demonstrated. The IR of both CM and DA was greater for older cows in early lactation (Figure 2). The IR of DI was greater for early lactation cows with parity 2 and ≥3. The occurrence of FD was greater for cows in parity 1 and 2 in late lactation (Figure 2).

### 3.5. Association of Antibiotic Treatments with Parity, Stage of Lactation, Season, and Bulk Tank Somatic Cell Count

For CM, ME, FD, DA, DI and PN, there were no associations between parity and the probability of receiving antibiotic treatments (*p* > 0.35). As compared to cows diagnosed with CM in early (reference) or mid-lactation (OR = 1.0), late lactation cows were less likely to receive antibiotics for treatment for CM (OR = 0.77 [95%CI 0.66–0.90]; *p* = 0.001). The odds of antibiotic treatment for FD differed among cows based on stage of lactation (*p* < 0.001). As compared to cows in early lactation (reference), the probability of antibiotic treatment was less for cows in mid lactation (OR = 0.62 [95%CI 0.45–0.86]) and least for cows in late lactation (OR = 0.36 [95%CI 0.27–0.50]). Cows diagnosed with DA in mid (OR = 0.45 [95%CI 0.22–0.90]) or late lactation (OR = 0.52 [95%CI 0.29–0.93]) had less chance of antibiotic treatment as compared to cows diagnosed in early lactation (reference; *p* = 0.016). There were no significant differences in the probability of receiving antibiotics for treatment of DI based on stage of lactation (*p* = 0.093). However, the probability of antibiotic treatment of cows diagnosed with PN was less for cows in mid (OR = 0.46 [95%CI 0.27–0.79]) as compared to early (reference) or later lactation (0.72 [95%CI 0.42–1.23]; *p* = 0.018).

Cows diagnosed with CM and RFM in summer had about twice the odds of receiving antibiotics as compared to cows diagnosed during other seasons (*p* = 0.011). Cows diagnosed with CM had 13.4 times greater odds of receiving antibiotics if they were located on a farm that had BTSCC ≥ 150,000 cells/mL (*p* = 0.023). No associations of antibiotic treatment with farm size (*p* = 0.419) or RHA (*p* = 0.480) were observed.

### 3.6. Association of Retention on Farm with Parity, Stage of Lactation, and Season

For all diseases, parity was associated with the odds of retention (remaining on the farm rather than being culled or dying) (*p* < 0.001) and the probability of retention was greatest for parity 1, less for parity 2 and least for parity ≥ 3 cows. For cows diagnosed with ME, as compared to parity 1 cows (85.5% retained), the odds of retention were 0.80 [95%CI 0.65–0.99] for cows in parity 2 (82.6% retained) and 0.33 [95%CI 0.28–0.39] for parity ≥ 3 cows (65.9% retained). For cows diagnosed with FD, as compared to parity 1 cows (88% retained), the odds of retention were 0.59 [95%CI 0.48–0.74] for cows in parity 2 (81.4% retained) and 0.24 [95%CI 0.20–0.29] for parity ≥ 3 (63.8% retained). For cows diagnosed with RFM, as compared to parity 1 cows (87.5% retained), the odds of retention were 0.66 [95%CI 0.51–0.85] for cows in parity 2 (82.2% retained) and 0.38 [95%CI 0.31–0.48] for parity 3+ (72.8% retained). For cows diagnosed with PN, as compared to parity 1 cows (47.9% retained), the odds of retention were 0.69 [95%CI 0.50–0.95] for cows in parity 2 (38.8% retained) and 0.45 [95%CI 0.33–0.61] for parity ≥ 3 (29.1% retained) respectively.

Stage of lactation was associated with the odds of retention for cows diagnosed with CM, FD, DA, DI, and PN (*p* < 0.001). For cows diagnosed with FD, as compared to cows in early lactation (84.3% retained), the odds of retention were 0.77 [95%CI 0.62–0.95] for cows in mid (80.5% retained) and 0.48 [95%CI 0.39–0.57] for cows in late lactation (71.9% retained). For cows diagnosed with PN, as compared to cows in early lactation (54.8% retained), the odds of retention were 0.35 [95%CI 0.26–0.49] for cows in mid (29.9% retained) and 0.38 [95%CI 0.28–0.52] for cows in late lactation (31.6% retained).

**Figure 3:**
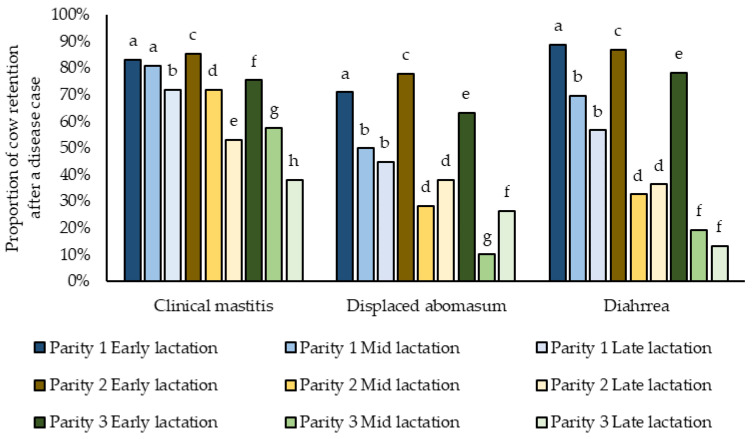
Frequency of retention in the herd after a disease case (clinical mastitis, displaced abomasum, and diarrhea) with interaction factor of parity and stage of lactation for 37 WI dairy herds in 2017. Different letters mean the comparison among stages of lactation within parity and disease were significant (*p* < 0.003).

For three diseases (CM, DA, and DI) our models for retention included significant interactions between parity and stage of lactation (Figure 3). For CM, first parity cows in early and mid-lactation were more likely to be retained, while the odds of retention decreased as lactation progressed for cows in 2nd and ≥3 parities. Regardless of parity, cows diagnosed with DA or FD in early lactation were more likely to be retained as compared to cows diagnosed in later stages of lactation (Figure 3).

For cows diagnosed with CM, ME, and RFM, the probability of retention was associated with season of diagnosis. As compared to cows diagnosed with CM in Fall (74.5% retained), cows were more likely to be culled in other seasons (odds ratios for retention were 0.86 [95%CI 0.77–0.96] for cases diagnosed in Winter (71.5% retained) and 0.70 [95%CI 0.63–0.79] for cases diagnosed in both Spring and Summer (67.2% retained; *p* < 0.001). Conversely, for cows diagnosed with ME, cows diagnosed in Winter (84.6% retained) were more likely to be retained as compared to Fall (OR = 1.76 [95%CI 1.40–2.23]; 75.6% retained; *p* < 0.001), but retention was similar based on diagnosis of ME in Fall, Spring or Summer. As compared to cows diagnosed with RFM in Fall or Summer (79% retained), the odds of retention within the herd were greater for cows diagnosed in Winter (OR = 1.53 [95%CI 1.17–1.99]; 85.2% retained) or Spring (OR = 1.26 [95%CI 0.99–1.59]; 82.6% retained).

## 4. Discussion

As dairy herds have expanded and modernized, the focus of health management in dairy cattle has successfully shifted from treatment to prevention [16]. Rapid consolidation of the U.S. dairy industry has dramatically changed herd structure and in 2017, 78% of milk produced in the U.S. came from herds that contained >200 milking cows [17]. While the frequency of some diseases in dairy farms has been reported in the context of other objectives [18], the IR of common diseases has not been reported for cows on large dairy farms that now produce most of the nation’s milk. Our objective was to describe the incidence and treatments of mastitis and other selected diseases for cows on larger farms using curated data from 37 large dairy farms in Wisconsin. While we based our estimates on electronic animal health records (rather than percent of cows affected), our estimates for CM, ME and RFM (the most commonly occurring diseases) were comparable to those reported in a national survey [1].

The population of herds included in our study is representative of well managed larger dairy farms in the Upper Midwest. Large herds (>250 lactating cows) in this region have previously been documented to have adopted many recommended best management practices [19]. The bulk tank SCC of only 1 herd exceeded 200,000 cells/mL (while 3 were less than 100,000 cells/mL) and average milk production was well above the US average of about 10,300 kg/cow [1]. As compared to earlier studies, improvement in udder health was evident, in the lower BTSCC we observed (average of 142,600 cells/mL) in comparison to that observed almost ten years ago in a similar population of larger WI dairy farms (average of 219,000 cells/mL) [20]. However, variation in disease incidence was evident among farms, and some farms presented with low BTSCC, but higher IR of mastitis. Some variation in IR may be related to variation in detection, case definitions or recording bias but these biases were minimized by the validation of detection and recording procedures during the interview researchers conducted during the farm visit. However, these results are representative of farmer recognized, diagnosed, and recorded disease events and it is likely that they underestimate the occurrence of some diseases.

Several studies have collected disease data from small or medium sized farms (<250 lactating cows). Recent studies of common bovine diseases showed similar IR of CM (24%), RFM (6.9%), DA (1.9%) and PN (1.4%) in the Northeast area in the USA, but higher frequencies of ME (22.8%), LA (10.7%) and KE (30.2%) were reported [21]. Discrepancies of frequencies of ME, LA and KE might be explained by use of diagnostic criteria (e.g., evaluation of vaginal discharge at 7 and 28 DIM for ME; collection of blood samples at 7 days in milk for determination of serum beta hydroxybutyrate (BHB) for KE; and 1 to 5 scoring system at 35 days in milk for LA) which varied among farms included in our study. With the exception of LA, our results were similar to rates described in a nationally representative survey [1], where CM affected 24.8% of cows per herd per year, LA affected 16.8%, ME 6.9%, RFM 4.5%, KE 4.2%, DI 3.2%, PN 2.8%, MF 2.8% and DA 2.2% of cows per herd per year.

While it is difficult to compare results of studies conducted in different geographic regions and herd sizes or using different sources of data, some diseases remain highly incident (CM, ME, and FD) while others appear to have been controlled in comparison to previous research [1,21]. As compared to previous studies, the IR of important metabolic diseases such as MF and KE have decreased which may be a consequence of improved management of diets by professionals who commonly balance rations on large dairy farms.

The dairy management software used by farms enrolled in this study is highly adopted throughout the U.S., but the data storage format for this program allows only limited characters, and remarks associated with UDE are customized for each farm. Thus, extraction and classification of UDE is a labor-intensive process that requires examination of remarks and consultation with farm managers to ensure accurate understanding of each event. Each of these farms was visited as part of the original study and remarks were reviewed with animal health managers. However, for some diseases (especially those that do not present obvious clinical signs), misclassification of some events is possible. Detection and recording biases are also possible, especially for diseases that may not be treated during lactation. For example, researchers evaluated cows for lameness using a 5-point locomotion scoring method and reported that the median prevalence of lameness was 18.3% [18]. Diseases such as displaced abomasum (reported in all farms), retained fetal membranes (reported in 94.6% of farms) and milk fever (reported in 86.5% of farms) present obvious clinical signs during early lactation (when cows are more frequently examined) and do not frequently recur thus our estimates for IR are likely more precise as compared to our estimates of IR for CM (requires recording observations of foremilk to detect mild cases) or ketosis (requires diagnostic testing) which require practices that may be more variably applied among farms. In general, use of producer diagnosed and recorded UDE reflects the apparent incidence of these diseases as perceived by producers.

Bacterial diseases are commonly treated using antimicrobials. Our data were collected as part of a study focused on quantifying antimicrobial usage on dairy farms and we previously reported considerable variation among farms in antimicrobial usage [11]. Similar variation in disease incidence among farms was observed in this analysis and only CM and DA were reported on all farms. Prevention of both bacterial diseases and metabolic diseases (such as MF and KE that increase risk of bacterial diseases) is necessary to successfully reduce antimicrobial usage. Clinical mastitis was the most common bacterial disease and the IR ranged from <1% (Farm 18) to >50% (Farm 28) per 100 cow-lactations. While both Farm 18 and Farm 28 contained about 1000 lactating cows and produced similar amounts of milk per cow, it was apparent that Farm 18 had greater emphasis on prevention of disease. The BTSCC is a good indicator of the prevalence of subclinical mastitis and the BTSCC of Farm 28 was 320,000 cells/mL in contrast to the BTSCC of Farm 18 which was 140,000 cells/mL. On Farm 18, the IR of all diseases was less than the median IR for all farms. In contrast on Farm 28, the IR exceeded the median for 6 of 9 diseases, even though this farm reported one of the greatest culling rates (48%). Use of antimicrobials on dairy farms is necessary to maintain welfare of some animals affected with bacterial infections but selection of antimicrobials must be done judiciously to reduce risks of selection for resistance and occurrence of antimicrobial residues in milk and meat [22]. Due to the frequency of occurrence, treatment and control of mastitis accounts for most doses of antimicrobials given to dairy cows, but depending on etiology, many of these cases will experience spontaneous cure without use of antimicrobials [23,24] and some chronically affected cows have low probability of achieving bacteriological cure even when antimicrobial are used [13]. Thus, reductions in antimicrobial usage are possible for many farms but emphasis must be placed on maintaining animal health and prevention of all diseases rather than treatment after it occurs.

Similar to previous reports [1,12], ceftiofur (a highest priority critically important antimicrobial [25]; CIA) was commonly used for treatment of bovine diseases. Restrictions in usage of “highest priority” CIAs in veterinary medicine have been recommended and some countries have enacted voluntary or legislated reductions in their use [26]. For example, use of 3rd and 4th generation cephalosporins (primarily ceftiofur) dropped from 18% to <1% of antimicrobial usage (AMU) on dairy farms in the Netherlands in response to legislation [27] and without apparent negative impacts on animal health [28]. In N. America, Quebec has restricted the use of ceftiofur as a first choice treatment in dairy cows [29]. In the U.S., such restrictions are controversial because of concerns about reducing therapeutic options for veterinarians who work with food-producing animals [30]. Ceftiofur is the most used antimicrobial on U.S. dairy farms [11,31] and is the only CIA approved for use in mature cows on US dairy farms [32]. As a 3rd generation cephalosporin that is similar to ceftriaxone, a drug used to treat serious Gram-negative infections in humans, concerns about emergence and dissemination of resistance via extended-spectrum beta-lactamases have been expressed [33,34,35,36,37]. In comparison to other approved antimicrobials, systemic treatments with ceftiofur do not require a milk-withdrawal period [11], thus reducing costs and the risk that poor quality milk from cows with mastitis is mingled with milk used for human consumption is increased. In our study, ceftiofur was used to treat most cases of PN, ME, and CM (usually IMM), and was used to treat >30% of cases of RFM. Previous researchers have reported that ME and RFM were frequently treated using penicillin, third generation cephalosporins, or a combination of ampicillin with oxytetracycline or cloxacillin [12,38,39].

For some diseases, additional education of producers could result in reduced usage of antimicrobials for treatment of diseases that have high rates of spontaneous cure. The effectiveness of intrauterine antimicrobial infusions for the treatment of cows with RFM remains questionable. For example, all antimicrobial treatments for RFM are extra-label since there are no drug with a label approval. For RFM, a key strategy is to avoid antimicrobial use by delaying treatment, as fetal membranes are usually naturally expelled in 32% of the cases or after light manual traction in 50% of the cases by 2 to 4 d postpartum RFM [12]. Interestingly, 36% of CM cases did not receive antimicrobials. This percentage has increased over the last ten years, likely due to increased adoption of selective treatment protocols based on use of on-farm culture. As these programs have been adopted, more farmers have understood the importance of avoiding antimicrobials in cases of spontaneous cure (e.g., when caused by non-severe coliforms cases such as *Escherichia coli*) or when the clinical case has no isolation of the causative agent (bacteriologically negative) [13].

Choices of treatment varied among dairy farms enrolled in our study. Some farms used very few antimicrobials regardless of disease event. For example, 92.1% of UDE recorded on Farm-40 had no associated antimicrobial treatments (Supplementary Table S1). In contrast, 84.2% of UDE recorded on Farm-21 received antimicrobial treatments. Interestingly, the IR rate of CM was similar on these farms. The variation that we observed is indicative of opportunities to improve disease detection and recording systems as well as reduce and refine antimicrobial usage for farms that used greater number of treatments.

Parity and stage of lactation were associated with IR of most diseases and emphasizes that early lactation continues to be an important period for implementation of preventive health practices [16]. We observed a greater risk of culling for older cows, and it is possible that older cows are preferentially removed from herds at a greater rate partially to reduce labor and costs associated with the greater risk of diseases in these animals. If improved cow retention is a goal, improved health management of older cows should be a priority. Increasing producer understanding of the risks associated with diseases not only serves to provide insight to producers for more informed culling decisions but may also help producers weigh the costs of adopting new methods and technologies targeted at reducing on-farm diseases [5].

Our study illustrates considerable variation in mastitis and other common diseases and treatments among large, productive dairy herds in Wisconsin. Some diseases remain highly incident such as mastitis, metritis and retained fetal membranes and treatments should be targeted based on known etiologies. Ceftiofur was widely used, but reductions are possible for cases that should be responsive to other approved antibiotics.

## 5. Conclusions

Most diseases occurred in older cows during early lactation and most treatments occurred during the same period. Younger cows affected with diseases in early lactation were more likely to be retained after diagnosis, as compared to older cows and cows in later stages of lactation. More than 30% of cows that had first cases of DA, CM, RFM, DI, and FD did not receive antibiotics, while more than 50% of cows diagnosed with PN, ME and CM were treated using ceftiofur. Health management programs on dairy farms should emphasize preventive practices, especially for higher risk cows and evidence-based treatment protocols that focus on judicious usage of antimicrobials should be emphasized. Antimicrobial therapy is used selectively for most diseases and the probability of receiving antimicrobial therapy varies among seasons, parities, and stage of lactation. Mastitis remains the most common disease of dairy cows and many older cows diagnosed with this disease are removed from production. Thus, mastitis control needs to be emphasized, even in herds with relatively low bulk tank somatic cell counts.

## Figures and Tables

**Table 1:** Descriptive information about antimicrobial treatments by active ingredient and routes for selected bovine diseases (not including KE & MF).

Treatment for Diseases	CM ^1^		ME SYS	RFM SYS	FD		DA SYS	PN SYS	DI SYS	UDE ^3^	
	IMM ^2^	SYS ^2^			SYS	TOP ^2^				n	Percent ^4^
Active ingredient											
Amoxicillin ^6,7^	0.6% ^5^	0.0%	0.0%	0.0%	0.0%	0.0%	0.0%	0.0%	0.0%	87	0.5%
Ampicillin	0.3%	1.7%	16.3%	17.2%	1.4%	0.0%	58.1%	11.0%	2.1%	2646	14.8%
Ceftiofur ^7^	52.8%	1.7%	68.6%	30.1%	2.4%	0.05	8.7%	71.6%	18.3%	13,225	74.1%
Cephapirin ^7^	5.5%	0.0%	0.0%	0.0%	0.0%	0.0%	0.0%	0.0%	0.0%	771	4.3%
Cloxacillin ^7^	0.0%	0.0%	0.0%	0.0%	0.0%	0.0%	0.0%	0.0%	0.0%	1	0.0%
Florfenicol ^6^	0.1%	0.1%	0.0%	0.0%	0.0%	0.0%	0.0%	0.6%	0.0%	18	0.1%
Hetacillin ^7^	2.0%	0.0%	0.0%	0.0%	0.0%	0.0%	0.0%	0.0%	0.0%	275	1.5%
Lincomyci ^6^	0.0%	0.0%	0.0%	0.0%	0.9%	0.9%	0.0%	0.0%	0.0%	46	0.3%
Oxytetracycline	0.2%	0.9%	2.2%	0.0%	0.4%	0.0%	0.1%	3.1%	0.0%	199	1.1%
Penicillin G ^7^	0.1%	0.2%	0.3%	0.1%	0.3%	0.0%	2.6%	0.0%	0.4%	97	0.5%
Pirlimycin ^7^	2.6%	0.0%	0.0%	0.0%	0.0%	0.0%	0.0%	0.0%	0.0%	359	2.0%
Spectinomycin ^6^	0.0%	0.02%	0.0%	0.0%	0.0%	0.0%	0.0%	0.0%	0.0%	2	0.0%
Sulfadimethoxine	0.0%	0.01%	0.1%	0.0%	0.0%	0.0%	0.2%	3.8%	0.1%	60	0.3%
Tetracycline	0.0%	0.0%	0.0%	0.0%	1.3%	1.3%	0.0%	0.0%	0.0%	67	0.4%
Tilmicosin ^8^	0.0%	0.0%	0.0%	0.0%	0.0%	0.0%	0.0%	0.2%	0.0%	3	0.0%
Tulathromycin ^8^	0.0%	0.0%	0.0%	0.0%	0.0%	0.0%	0.0%	0.1%	0.0%	1	0.0%
No antibiotic	35.9%		12.5%	52.6%	93.3%		30.3%	9.8%	79.0%	17,872	-

^1^ CM = clinical mastitis (n = 13,997); ME = metritis (n = 4948); RFM = retained fetal membranes (n = 3538); FD = foot disorders (n = 5176); DA = displaced abomasum (n = 1648) antimicrobials were not the primary treatment; PN = pneumonia (n = 1277); and DI = diarrhea (n = 1126); ^2^ Route of the medication: IMM = intramammary; SYS = systemic; and TOP = topical; ^3^ User-defined events; ^4^ Antimicrobial treatment as a proportion of total cases receiving antibiotics; ^5^ Percent of UDE receiving the antimicrobial; ^6^ No antimicrobials are approved for systemic treatment of CM in dairy cows, but restricted usage allowed under extra-label usage guidelines; ^7^ Approved intramammary formulation is available; ^8^ Not approved for use in lactating dairy cows (animal > 20 months of age).

**Table 2:** Incidence Rates (LSM) by parity group and stage of lactation for 35,729 cows on 37 Wisconsin farms in 2016–2017.

Selected Diseases	No. of Farms	Parity ^1^				Stages of Lactation ^2^			
		1	2	≥3	*p*-Value	Early	Mid	Late	*p*-Value
Clinical Mastitis ^3^	37	3.64% ^c^	6.81% ^b^	9.64% ^a^	<0.001	8.07% ^a^	5.80% ^b^	5.16% ^b^	<0.001
Metritis	33	9.8% ^a^	6.4% ^c^	7.9% ^b^	<0.001	-	-	-	-
Foot disorders ^3^	30	1.4% ^c^	2.0% ^b^	3.2% ^a^	<0.001	1.8% ^b^	1.8% ^b^	2.7% ^a^	<0.001
Retained fetal membranes	35	3.7% ^c^	4.9% ^b^	7.4% ^a^	<0.001	-	-	-	-
Displaced abomasum ^3^	37	0.5% ^b^	0.9% ^a^	1.1% ^a^	<0.001	2.0% ^a^	0.4% ^b^	0.6% ^b^	<0.001
Diahrrea ^3^	17	0.5%	0.6%	0.7%	0.180	1.1% ^a^	0.5% ^b^	0.4% ^b^	0.028
Pneumonia	35	0.7% ^b^	0.9% ^a^	1.1% ^a^	0.006	1.4% ^a^	0.7% ^b^	0.8% ^b^	<0.001
Ketosis	30	2.6% ^b^	2.9% ^b^	8.2% ^a^	<0.001	-	-	-	-
Milk fever	32	0.1% ^c^	0.7% ^b^	2.8% ^a^	<0.001	-	-	-	-

^1^ Parity 1 (n = 9424), parity 2 (n = 10,251) and parity 3+ (n = 16,054); ^2^ Stages of lactation: early (1–100 days; n = 23,405), mid (101–200 days; n = 5926) and late (>200 days; n = 6398); ^3^ For these diseases there were interactions between parity and stage of lactation; Parity and stage of lactation were forced in all models; Metritis, retained fetal membranes, ketosis and milk fever only occurred in early lactation; Different superscripts within rows (within parity and within lactation stage) indicates statistically significant differences.

## Data Availability

Restrictions apply to the availability of these data. Data was obtained from private dairy farms and are only available from the authors with the permission of each farmer.

## Supplementary Materials

The following supporting information can be downloaded at: https://www.mdpi.com/article/10.3390/pathogens11111282/s1, Table S1: Farm level descriptors of 37 Wisconsin dairy farms and the incidence rate of selected bovine diseases.
