# Supplementary material for: Incidence and Treatments of Bovine Mastitis and Other Diseases on 37 Dairy Farms in Wisconsin
Source: Pathogens. 2022 Nov 1;11(11):1282. doi: 10.3390/pathogens11111282 (PMC9698317; doi:10.3390/pathogens11111282)
Supplement: Supplementary file 1 [file pathogens-11-01282-s001.zip › pathogens-1969377-supplementary.pdf]

Supplementary Table S1. Farm level descriptors of 37 Wisconsin dairy farms and the incidence rate of selected bovine diseases.

**Farm**	**RHA** [1]	**Cow-lactations-year**				Parity			BTSCC [2]	Antibiotic treatments [3]	Incidence rate [4]								
		**Total**	Culled	Died	% Culled or died	1	2	≥3			CM	ME	RFM	KE	FD	DA	PN	MF	DI
1	13,141	2763	880	120	36.2	1045	817	901	122	21.4	44.3	6.4	5.5	16.5	57.7	2.2	7.7	1.3	2.6
2	14,878	474	162	14	37.1	194	137	143	80	18.1	29.9	7.2	8.9	7.0	88.1	5.3	0.2	0.4	5.3
3	13,313	593	102	125	38.3	208	150	235	175	35.0	1.7	9.4	3.0	8.3	34.6	6.2	2.9	0.7	-
4	13,608	1414	272	143	29.3	520	425	469	110	95.8	33.9	15.8	15.4	1.6	4.1	4.5	9.4	26.1	-
5	14,389	358	133	8	39.3	143	95	120	78	49.2	39.9	36.8	8.4	16.5	5.9	5.9	0.6	1.4	2.5
6	13,103	797	272	47	40.0	295	216	286	120	40.6	25.3	5.4	3.0	13.7	25.7	2.3	1.9	0.4	-
7	12,928	441	162	10	39.0	154	123	164	86	83.8	8.4	18.4	4.3	31.5	0.9	0.7	1.4	2.3	0.9
8	13,140	2405	733	125	35.7	888	676	841	187	92.4	20.4	4.7	3.0	1.5	3.4	2.7	1.6	0.3	0.1
9	10,829	2163	922	22	43.6	844	667	652	195	89.0	27.7	12.9	1.7	-	0.05	2.2	0.9	0.0	0.05
10	14,061	618	203	20	36.1	267	183	168	105	44.1	25.6	0.2	2.1	-	-	0.5	0.3	-	-
11	12,505	1129	265	78	30.4	382	334	413	181	92.4	26.1	8.7	12.0	-	1.1	6.2	3.1	1.8	-
12	13,298	501	152	19	34.1	166	149	186	121	57.5	15.2	7.8	15.4	17.0	36.9	3.8	6.4	0.6	-
13	14,075	2150	761	97	39.9	940	657	553	188	31.6	33.8	14.2	30.8	1.2	35.3	4.7	2.0	0.2	-
14	13,381	304	112	41	50.4	112	90	102	126	71.4	21.1	17.5	15.8	-	-	0.3	0.7	-	-
15	14,334	774	242	34	35.6	317	189	268	155	49.0	16.7	13.3	7.2	0.1	0.8	0.1	0.6	0.3	0.5
16	14,375	641	205	38	37.9	238	170	233	146	68.8	7.8	-	3.0	7.2	1.6	2.2	-	0.6	-
17	12,993	2208	911	68	44.3	932	704	572	118	32.5	17.4	3.1	5.8	6.4	8.5	1.2	1.3	1.8	-
18	13,608	1094	384	37	38.5	405	299	390	140	73.1	0.7	2.4	2.4	-	-	0.5	0.1	-	-
19	14,742	754	192	40	30.8	275	192	287	162	100.0	1.9	-	5.7	-	-	0.5	-	0.5	-
20	14,061	598	183	29	35.5	222	165	211	115	83.0	31.3	8.9	0.3	0.7	3.0	2.0	6.7	1.3	5.7
21	13,154	3247	1048	101	35.4	1144	828	1275	120	84.2	41.8	5.1	8.5	0.6	5.9	2.4	0.4	3.1	-
22	10,905	5211	1553	341	36.4	1999	1594	1618	128	93.4	33.8	6.4	0.8	2.3	2.2	3.6	1.0	0.9	-
23	13,337	1126	302	0	26.8	393	331	402	142	55.0	28.4	7.0	5.1	24.2	15.1	3.1	2.1	2.4	0.8
24	12,353	839	182	39	26.3	248	288	303	133	85.6	13.2	1.8	-	0.2	-	3.8	2.3	1.5	0.5
25	13,117	712	188	52	33.7	271	203	238	150	49.8	22.2	9.7	3.4	1.7	50.8	1.3	0.6	0.3	-
26	13,517	588	162	27	32.2	227	172	189	105	61.8	18.7	15.5	13.8	12.1	19.1	3.9	7.1	2.0	0.3
27	15,059	573	169	35	35.6	228	146	199	174	99.6	34.9	-	5.4	1.6	-	3.1	1.7	1.0	-
28	13,517	1030	413	78	47.6	409	309	312	320	94.4	53.4	29.5	9.0	11.1	4.4	3.5	3.1	1.3	-
29	14,288	623	275	27	48.4	248	183	192	160	95.3	22.6	8.3	11.4	11.2	2.6	3.5	13.5	-	-
30	14,601	366	96	29	34.1	136	83	147	188	97.1	13.6	0.8	-	1.6	0.5	1.4	2.7	-	-
31	13,608	1419	448	53	35.3	524	385	510	164	87.6	27.8	10.4	7.7	-	-	4.2	1.8	0.3	-
32	13,081	921	237	79	34.3	330	313	278	165	67.5	35.9	7.5	1.3	4.9	8.4	4.9	2.9	1.6	2.6
33	13,381	1335	454	94	41.0	520	481	334	148	78.7	13.3	-	8.9	1.9	0.1	2.9	1.3	0.4	0.2
34	13,245	3759	1087	87	31.2	1299	1340	1120	77	78.4	8.9	15.2	7.1	1.2	0.4	1.9	0.2	2.1	0.2
35	13,608	1196	451	84	44.7	461	282	453	129	79.7	16.5	16.6	5.9	7.9	9.3	8.5	5.9	1.3	0.7
36	12,530	1621	618	56	41.6	707	436	478	137	31.8	43.8	29.1	9.3	36.2	3.9	3.3	4.0	1.0	49.6
37	10,886	3580	1109	190	36.3	1333	1198	1049	126	7.9	46.8	13.7	8.7	10.9	3.6	6.5	4.0	1.4	3.2

[1] Rolling herd average for milk (kg per cow per year); [2] Bulk tank SCC ($\times 10^3$ cells/mL) from the month preceding farm visit; [3] Percentage of all disease events were treated with an antibiotic during one retrospective year; [4] Cases per 100 cow-lactations-year: CM = clinical mastitis (n = 13,997 user-defined events); ME = metritis (n = 4948); RFM = retained fetal membranes (n = 3538); KE = ketosis (n = 3090); FD =feet disorders (n = 5176); DA = displaced abomasum (n = 1648); PN = pneumonia (n = 1277); MF = milk fever (n = 929); and DI = diarrhea (n = 1126); Dash indicates disease was not recorded in the farm computer-based records.
